# Intervention in gut microbiota increases intestinal γ-aminobutyric acid and alleviates anxiety behavior: a possible mechanism via the action on intestinal epithelial cells

**DOI:** 10.3389/fcimb.2024.1421791

**Published:** 2024-09-05

**Authors:** Mion Ikegami, Hikari Narabayashi, Kazuaki Nakata, Miyu Yamashita, Yutaka Sugi, Yushiro Fuji, Hiroshi Matsufuji, Gaku Harata, Kazutoyo Yoda, Kenji Miyazawa, Yusuke Nakanishi, Kyoko Takahashi

**Affiliations:** ^1^ Nihon University Graduate School of Bioresource Sciences, Fujisawa, Kanagawa, Japan; ^2^ College of Bioresource Sciences, Nihon University, Fujisawa, Kanagawa, Japan; ^3^ Technical Research Laboratory, Takanashi Milk Products Co., Ltd, Yokohama, Kanagawa, Japan

**Keywords:** γ-aminobutyric acid, gut microbiota, intestinal epithelial cell, probiotics, gut-brain axis

## Abstract

The role of the gut microbiota in the gut-brain axis has attracted attention in recent years. Some gut microbiota produces γ-aminobutyric acid (GABA), a major inhibitory neurotransmitter in mammals, *in vitro*, but the correlation between gut microbiota composition and intestinal GABA concentration, as well as the action of intestinal GABA *in vivo*, are poorly understood. Herein, we found that the intestinal GABA concentration was increased in mice by the intervention of the gut microbiota with neomycin or *Bifidobacterium bifidum* TMC3115 (TMC3115). Administration of TMC3115 reduced anxiety without affecting serum levels of serotonin, corticosterone, or GABA. We further found that intestinal epithelial cells expressed GABA receptor subunits and mediated mitogen-activated protein kinase signaling upon GABA stimulation. In addition, administration of TMC3115 induced mitogen-activated protein kinase signaling in colonic epithelial cells but not in small intestinal epithelial cells in mice. These results indicate that GABA produced by the gut microbiota, mainly in the colon, may affect host behavioral characteristics via GABA receptors expressed in intestinal epithelial cells without being transferred to the blood. This study suggests a novel mechanism by which intestinal GABA exerts physiological effects, even in the presence of the blood-brain barrier.

## 1 Introduction

Numerous commensal bacteria inhabit the intestinal lumen, forming the gut microbiota, and exert various physiological effects on the host. Gut microbiota have been shown to play a crucial role in host health as it enhances resistance to pathogens (Wahi et al., 2024) and promotes the maturation and development of the intestinal immune system (Round and Mazmanian, 2009). In contrast, dysbiosis, a disturbance in the diversity of the gut microbiota, has been reported to correlate with various diseases, such as allergies, cancer, and diabetes (Noverr and Huffnagle, 2005; Sadrekarimi et al., 2022; Gomes et al., 2018). Recently, the correlation between gut microbiota and neurological and psychiatric disorders with social and emotional problems, which are increasing in number, has attracted attention from the perspective of the gut-brain axis. The composition of the gut microbiota differs between patients with depression and healthy controls (Colomer et al., 2019), and the transplantation of the microbiome from patients with depression into germ-free recipient mice induces depression-like behaviors (Liu et al., 2021). Moreover, the lack of a microbiome leads to hyperactivity and deficits in stress response and social behavior (Bercik et al., 2011; Sudo et al., 2004; Desbonnet et al., 2014; Buffington et al., 2016) and specific gut microbes regulate neurotransmitter concentrations, such as serotonin, in the gut and serum (Yano et al., 2015), suggesting that the gut microbiota possesses a substantial action against neurological and psychiatric disorders.

Although the mechanisms underlying the effects of gut microbiota on neurological and psychiatric disorders have not been fully elucidated, gut microbe-derived metabolites are considered to play a key role (Needham et al., 2020; Spichak et al., 2021; Foster, 2022). Some metabolites produced by the gut microbiota have been reported to regulate not only intestinal functions but also brain and neuronal functions. Butyrate strengthens the intestinal epithelial barrier by inducing Mucin 2 expression in goblet cells (Hatayama et al., 2007) and ameliorates social abnormalities by modulating neurotransmitter gene expression in the frontal cortex via histone deacetylase inhibition (Kratsman et al., 2016). Furthermore, a mouse model of autism spectrum disorder exhibits increased intestinal permeability and elevated systemic levels of 4-ethylphenyl sulfate (4-EPS) derived from a precursor produced by the gut microbiota (Hsiao et al., 2013). 4-EPS has been shown to impair oligodendrocyte maturation in the brain, thereby causing anxiety-like behaviors, which can be restored by controlling 4-EPS levels following the administration of *Bacteroides fragilis* (Needham et al., 2022). Other metabolites derived from gut microbiota, such as lactate, tryptophan metabolites, and secondary bile acids, also affect sociability, emotion, and cognitive functions in mice and humans (Ortega et al., 2022).

In this study, we focused on γ-aminobutyric acid (GABA), a major inhibitory neurotransmitter in mammals known to exert various physiological effects, including tranquilizing (Lydiard, 2003), antistress (Dolfen et al., 2021), antihypertensive (Hayakawa et al., 2002), and immunomodulatory (Jin et al., 2013) activities. Some gut microbes, particularly Lactobacillus and Bifidobacterium, such as *L. brevis, L. plantarum, B. dentium*, *B. adolescentis*, and *B. angulatum*, have been reported to produce GABA when cultured *in vitro* (Barrett et al., 2012; Yunes et al., 2016; Li et al., 2010), suggesting that intestinal GABA concentrations depend on production by the gut microbiota as well as food-derived GABA. However, little is known about the correlation between intestinal GABA concentration and microbiota composition *in vivo* or the function of intestinal GABA in host physiology and its underlying mechanisms. Although it is currently understood that the action of GABA is mediated through receptors expressed abundantly in the brain, it may be difficult to explain the various activities of GABA by this mechanism alone. Moreover, as a consensus that GABA penetrates the blood-brain barrier (BBB) has not been established, it remains to be elucidated whether intestinal GABA reaches the brain after its transfer to the blood.

Two types of GABA receptors (GABARs), the ion channel GABA A receptor (GABA_A_R) and the G-protein-coupled GABA B receptor (GABA_B_R), are abundantly expressed in the brain tissue. In contrast, the expression and function of GABA receptors in tissues other than the brain, particularly in non-neuronal cells, are largely unknown, except for a few reports on neutrophils and airway smooth muscles (Rane et al., 2005; Osawa et al., 2006; Mizuta et al., 2007). Herein, we hypothesized that microbiota-derived intestinal GABA may act through receptors expressed on intestinal epithelial cells (IECs) that line the mucosal surface and are constantly exposed to luminal contents, including microbial metabolites. To test this hypothesis, we investigated the correlation between intestinal GABA concentration and microbiota composition, as well as the effects of intestinal GABA on host behavior and its underlying mechanisms, focusing on intracellular signals elicited via GABA receptors on IECs.

## 2 Materials and methods

### 2.1 Mice

Six-week-old female BALB/cA mice were purchased from CLEA Japan (Tokyo, Japan) and maintained in a temperature-controlled room with a 12-hour light/dark cycle and free access to food and water. Experiments involving fecal and blood samples and behavioral tests were conducted during the light cycle. All experiments were approved by the Nihon University Animal Care and Use Committee and were conducted in accordance with their guidelines.

### 2.2 Administration of antibiotics

**Figure 1:**
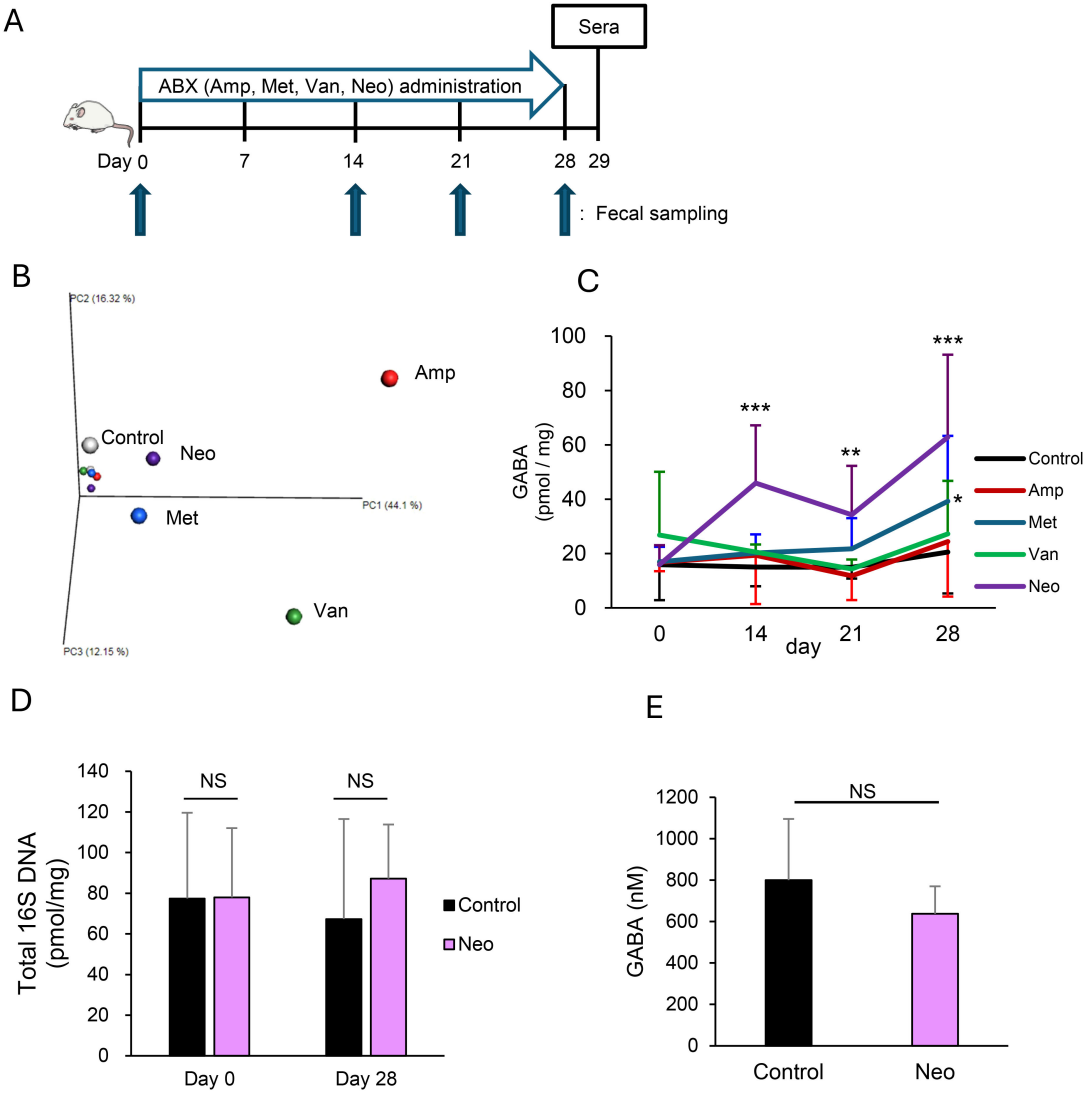
Neomycin treatment increases intestinal γ-aminobutyric acid (GABA) concentration through changes in the gut microbiota composition. **(A)** Experimental design of antibiotic (ABX) treatments. Mice were treated orally with ampicillin (Amp), metronidazole (Met), vancomycin (Van), or neomycin (Neo) in drinking water for four weeks. **(B)** Compositions of gut microbiota in Amp, Met, Van, and Neo-treated and untreated (Control) mice were determined from 16S rRNA sequences using fecal DNA pooled from 3 mice/group on days 0 and 28. The principal coordinate analysis plot of β-diversity is shown. Each group is indicated in a different color: Control, white; Amp, red; Met, blue; Van, green; Neo, purple. Small and large dots represent the data on days 0 and 28, respectively. **(C)** Fecal GABA concentration was measured using an ultra-performance liquid-chromatography-tandem mass spectrometry (UPLC-MS/MS) system in the control (n = 15), Amp (n = 6), Met (n = 12), Van (n = 6), and Neo (n = 18) groups on days 0, 14, 21, and 28. Data are presented as mean ± SD. *P < 0.05; **P < 0.01; ***P < 0.001 vs. Control. **(D)** Total amount of 16S rRNA genes in feces was measured using qPCR in the Control (n = 6) and Neo (n = 6) groups on days 0 and 28. Data are presented as mean ± SD. NS, not significant. **(E)** Serum GABA concentrations were measured using the UPLC-MS/MS system in the Control (n = 12) and Neo (n = 12) groups on day 29. Data are presented as mean ± SD. NS, not significant.

After one week of acclimatization, mice were divided into five groups to start administration (day 0): a control group and experimental groups administrated with ampicillin sodium (1 g/L, Wako, Osaka, Japan), vancomycin hydrochloride (0.5 g/L, Sigma, St. Louis, MO, USA), metronidazole (0.5 g/L, Sigma), and neomycin trisulfate salt hydrate (1 g/L, Sigma). Antibiotics were administered via drinking water for four weeks. Feces were collected on days 0 (before administration), 14, 21, and 28, and blood was collected on day 29. The experimental schedule is schematically shown in **Figure 1A**.

### 2.3 Administration of probiotics

**Figure 2:**
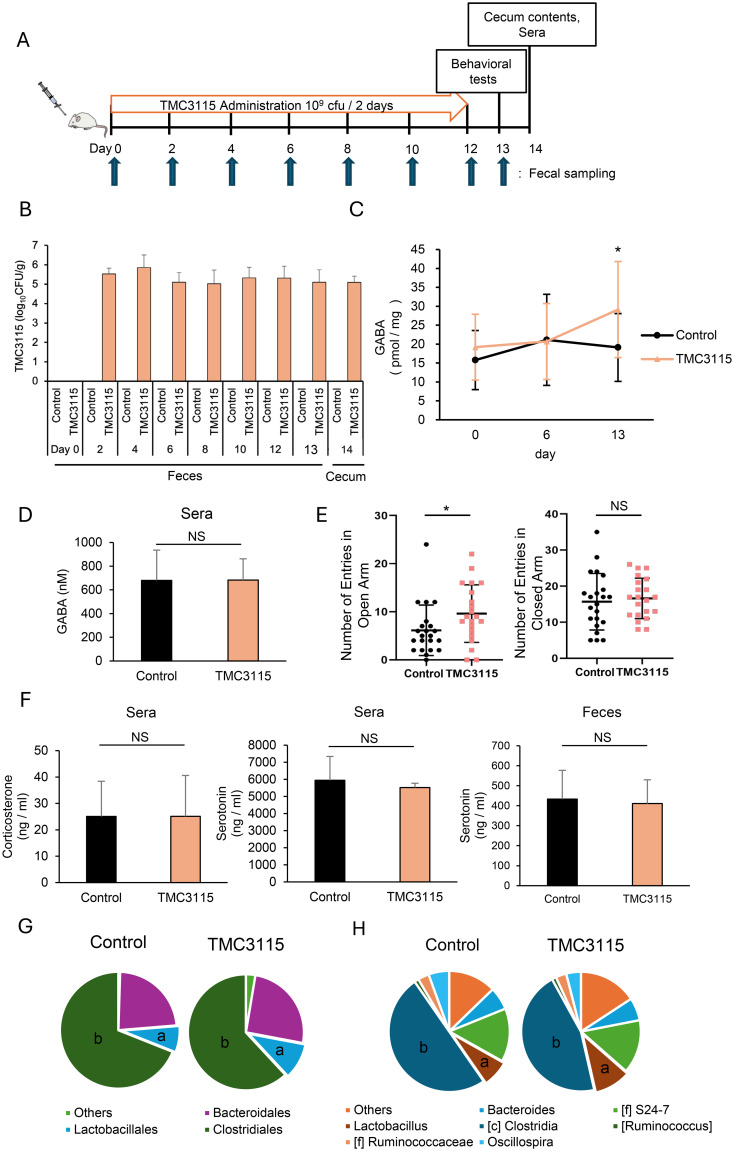
*Bifidobacterium bifidum* TMC3115 (TMC3115) increases intestinal γ-aminobutyric acid (GABA) concentration and alters mice behavior through changes in the gut microbiota composition. **(A)** Experimental design of TMC3115 administration. **(B)** TMC3115 in feces and cecal contents of the Control (n = 16) and TMC3115 (n = 15) groups was quantified using qPCR. Data are presented as mean ± SD. **(C)** Fecal GABA concentration was measured using ultra-performance liquid-chromatography-tandem mass spectrometry (UPLC-MS/MS) system in the Control (n = 16) and TMC3115 (n = 15) groups on days 0, 6, and 13. Data are presented as mean ± SD. *P < 0.05 vs. Control. **(D)** Serum GABA concentration was measured using UPLC-MS/MS system in the Control (n = 5) and TMC3115 (n = 5) groups on day 13. Data are presented as mean ± SD. NS, not significant. **(E)** Numbers of entries in open (left) and closed (right) arms in elevated plus maze test are shown for the Control (n = 22) and TMC3115 (n = 21) groups. Individual data and mean ± SD are presented. NS, not significant; *P < 0.05. **(F)** Serum corticosterone (left), serum serotonin (middle), and fecal serotonin (right) were measured using ELISA in the Control (n = 6–11) and TMC3115 (n = 5–10) groups on day 13 (fecal) or 14 (serum). Data are presented as mean ± SD. NS, not significant. **(G, H)** Bacterial occupancies at order **(G)** and genus **(H)** levels were analyzed using 16S metagenomic sequence in the Control (n = 5) and TMC3115 (n = 5) groups using cecal contents on day 14. Mean values are presented using pie charts of bacteria with more than 1% occupancies. a, Lactobacillales **(G)** and Lactobacillus **(H)**; b, Clostridiales **(G)** and Clostridia **(H)**.

After one week of acclimatization, the mice were divided into three groups to start administration (day 0): the control group was administered saline, and the experimental groups were administered *Bifidobacterium bifidum* TMC3115 (TMC3115) or *Lactobacillus rhamnosus* GG (LGG). Lyophilized TMC3115 and LGG was suspended in saline and administered at 10^9^ CFU/head/day by oral gavage every two days for two weeks. Feces were collected on days 0 (before administration), 2, 4, 6, 8, 10, 12, and 13, and an elevated plus maze test was conducted on day 13. After the administration period, blood was collected, and the medial small intestine, colon, and cecum were surgically removed from the mice on day 14. The experimental schedule is schematically shown in **Figure 2A**.

### 2.4 Determination of GABA concentration in fecal and blood samples

Fecal samples collected on days 0, 14, 21, and 28 (antibiotics administration experiment) and on days 0, 6, and 13 (probiotics administration experiment) were suspended with hot water (95-98°C) at the concentration of 0.1 g/mL and sonicated for 15 min. After centrifugation at 15,000 rpm at 4°C for 15 min, the supernatants were filtrated through 0.20-μm DISMIC-13CP (ADVANTEC, Tokyo, Japan). Sera were prepared from the blood, purified using acetone precipitation, and concentrated using a centrifugal evaporator. Fecal and serum samples were derivatized using the AccQ-Taq Ultra Derivatization Kit (Waters, Milford, MA, USA) for ultra-performance liquid-chromatography-tandem mass spectrometry (UPLC-MS/MS) analysis using a Xevo TQ-S mass spectrometer coupled to an ACQUITY UPLC system (Waters). An AccQ-Tag Ultra Column (2.1×100 mm, 1.7 µm, Waters) was used for UPLC under the following conditions: column temperature, 55°C; sample injection volume, 1 μL; and flow rate, 0.7 mL/min. The mobile phase consisted of gradient mixture of AccQ-tag Ultra Eluent A and B (Waters) as follows: 0−0.54 min, 0.1% B; 0.54−5.74 min, 0.1−9.1% B; 5.74−7.74 min, 9.1−21.2% B; 7.74−8.04 min, 21.2−59.6% B; 8.04−8.05 min, 59.6−90% B; 8.05−8.64 min, 90−90% B; 8.64−8.73 min, 90−0.1% B; and 8.73−9.50 min, 0.1−0.1% B. Mass spectra were acquired using positive electrospray ionization, and GABA was detected using multiple reaction monitoring. The ion source conditions were optimized as follows: source temperature, 150°C; capillary voltage, 0.5 kV; desolvation temperature, 500°C; flow rate of desolvation gas, 1000 L/h; and flow rate of cone gas, 50 L/h. Specific transitions of parent and product ions were monitored under the following conditions: MRM transition, 274>171; cone voltage, 20 V; and collision energy, 35 eV. Serially diluted GABA standard solution from 100 to 50000 fmol/μL was similarly derivatized and analyzed for the calibration curve. The molar concentrations of GABA in serum and per gram of feces were calculated.

### 2.5 Microbiome analysis

DNA was extracted from fecal samples and cecum contents using a QIAmp DNA stool kit (Qiagen, Venlo, Nederland) or NucleoSpin^®^ DNA Stool kit (MACHEREY-NAGEL, Dueren, Germany) according to the manufacturers’ instructions. The DNA concentration was analyzed using a Quantus Fluorometer (Promega, Madison, WI, USA) and the Quantifluor dsDNA System (Promega). The sequencing library was constructed using index-appended amplicons of the V3–V4 region of the 16S rRNA gene according to the 16S metagenomic sequencing library preparation manual (Illumina, San Diego, CA, USA). A MiSeq Reagent Kit v3 (600 cycles) (Illumina) was used for paired-end sequencing on the MiSeq platform (Illumina). Data analysis was performed using the QIIME pipeline. Next-generation sequencing data have been deposited in the DDBJ Sequence Read Archive (https://www.ddbj.nig.ac.jp/dra) under accession numbers PRJDB17874 and PRJDB17882.

### 2.6 Quantification of total intestinal bacteria and TMC3115

The amount of total intestinal bacteria was determined by quantifying the DNA encoding the 16S rRNA gene in feces on days 0 and 28 of the antibiotic administration experiment using quantitative PCR (qPCR). DNA concentrations per gram of feces were calculated from a standard curve using serially diluted 16S rRNA gene PCR amplicon with known concentration. TMC3115 was quantified by qPCR using DNA prepared from fecal samples collected on days 0, 2, 4, 6, 8, 10, 12, and 13, as well as cecum contents. CFU per gram of feces was calculated from a standard curve using DNA prepared from TMC3115 with known CFU. The nucleotide sequences of the primers used in the experiment were as follows:

16S rRNA gene:
Forward, 5′-CCTACGGGNGGCWGCAG-3′; Reverse, 5′-GACTACHVGGGTATCTAATCC-3′ (N, W, H, and V represent A/C/G/T, A/T, A/C/T, and A/C/G, respectively).
TMC3115:
Forward, 5′-GGAAAGCACGAAGACACACA-3′; Reverse, 5′-GCCGTCTGTTTGAAGAAACC-3′.

### 2.7 Elevated plus maze test

Anxiety-like behaviors were examined using the elevated plus maze test. The apparatus consisted of four arms (30 cm length, 6 cm width), two of which were closed by 15 cm high walls, and the other two were open without walls. Their arms were crossed 40 cm above the ground to create a positive shape. Mice were placed in the central area of the maze, facing one of the open or closed arms at the same frequency between the groups, and allowed to explore freely. The sessions were recorded for 5 min. The number of entries into the open or closed arms was analyzed by tracking the position of the mouse using the ANYMAZE software (Stoelting, Wood Dale, IL, USA).

### 2.8 Determination of serum corticosterone concentration

Corticosterone concentrations in the sera were measured using a Corticosterone ELISA Kit (Cayman Chemical Company, Ann Arbor, MI, USA) according to the manufacturer’s instructions.

### 2.9 Determination of fecal and serum serotonin concentration

Fecal extracts were prepared on day 13 of the TMC3115 administration experiment, as described above. Serotonin concentrations in the fecal extracts and sera were measured using a Serotonin ELISA Kit (Enzo Life Sciences, Farmingdale, NY, USA) according to the manufacturer’s instructions.

### 2.10 Preparation of murine IECs

IECs were prepared from the medial small intestine and colon of mice as previously described (Sugi et al., 2016). Briefly, after removing the Peyer’s patches, the intestinal tissues were washed with Ca^2+^-, Mg^2+^-free Hank’s balanced salt solution (Sigma) containing 5% fetal bovine serum (Capricorn Scientific, Land Hessen, Germany), 1 mM dithiothreitol, and 0.5 mM EDTA with shaking. Single-cell suspensions were obtained by treatment with dispase (BD Biosciences, Franklin Lakes, NJ, USA) to deplete lymphocytes by magnetic-activated cell sorting separation using a biotin-conjugated anti-CD45 antibody (eBioscience, San Diego, CA, USA) coupled with a Dynabeads biotin binder (Invitrogen, Thermo Fisher Scientific, Waltham, MA, USA).

### 2.11 Preparation of murine organoids

Organoids were established from crypts isolated from the small intestines of mice using Intesticult™ Organoid Growth Medium (STEMCELL Technologies, Vancouver, Canada) according to the manufacturer’s instructions. Briefly, the small intestines were opened longitudinally, cut into 2-mm segments, and washed 15–20 times with cold phosphate-buffered saline. The intestinal segments were incubated with the Gentle Cell Dissociation Reagent (STEMCELL Technologies) on a rocking platform to dissociate the crypts. The crypts were purified using a stepwise filtration through 70-μm strainers, resuspended in a 3:7 mixture of Intesticult™ Organoid Growth Medium and Matrigel^®^ Growth Factor Reduced Basement Membrane Matrix Phenol Red-Free (Corning, Corning, NY, USA), and seeded in 24-well plates at 20,000 crypts/well. After the Matrigel solidified, Intesticult™ Organoid Growth Medium was added to the wells to initiate the culture. The medium was replaced three times per week.

### 2.12 Human IEC lines

Human colonic adenocarcinoma cell lines Caco-2 and HT-29 and colon carcinoma cell line HCT116 were purchased from Summit Pharmaceuticals International (Tokyo, Japan). The cells were cultured as previously described (Narabayashi et al., 2022).

### 2.13 Determination of gene expression using reverse transcription-qPCR

Total RNA was extracted from IECs and organoids using the High Pure RNA Isolation Kit (Roche, Basel, Switzerland) and RNeasy Micro Kit (Qiagen, Venlo, Netherlands), respectively. Total RNA was reverse-transcribed using Super-Script IV reverse transcriptase and oligo(dT)_18_ primers (Invitrogen). The resulting cDNA and mouse brain cDNA purchased from BioChain (Newark, CA, USA) were used as templates for qPCR with CFX96 Touch Real Time PCR Detection System (Biorad, Hercules, CA, USA) using the KAPA SYBR^®^ Fast qPCR kit (KAPA Biosystems, Wilmington, MA, USA). The expression of each GABAR subunit was normalized to that of *Gapdh*. Nucleotide sequences of the primers used in this study are listed in **Table 1**.

**Table 1:** Primers used for determination of GABAR expression by qPCR.

Species	Target gene	Primer sequence
*human*	*GABA_A_R ß1*	F: 5’-GTACAAAATCGAGAGAGTCTGGG-3’ R: 5’-GCGAATGTCATATCCTTTGAGCA-3’
	*GABA_A_R ß2*	F: 5’-GCAGAGTGTCAATGACCCTAGT-3’ R: 5’-TGGCAATGTCAATGTTCATCCC-3’
	*GABA_A_R ß3*	F: 5’-CAAGCTGTTGAAAGGCTACGA-3’ R: 5’-ACTTCGGAAACCATGTCGATG-3’
	*GABA_B1_R*	F: 5’-CAGATAAATGGATTGGAGGGT-3’ R: 5’-GAGAACTGAGACGGAGATAAAGAG-3’
	*GABA_B2_R*	F: 5’-CTCGAACATGCAAAGATCCTATAGAAG-3’ R: 5’-GAAGGAGGGTGGCACATGTCT-3’
*mouse*	*GABA_A_R ß1*	F: 5’-TATTCTCCTCAGCACCCTG-3’ R: 5’-CGAGTACATGGTGGCCTTGG-3’
	*GABA_A_R ß2*	F: 5’-AACTACATCTTCTTTGGGAGAGGA-3’ R: 5’-GGTCCATCTTGTTGACATCCAG-3’
	*GABA_A_R ß3*	F: 5’-GAGCACCGTCTGGTCTCCAGGA-3’ R: 5’-CGATCATTCTTGGCCTTGG-3’
	*GABA_B1_R*	F: 5’-CTGCCCGGATGTGGAACCTTA-3’ R: 5’-TCAGCATACCACCCGATGAGA-3’
	*GABA_B2_R*	F: 5’-TCCATCATGGGCCTCATG-3’ R: 5’-TCGCAGGTCCAGGAAGTA-3’
*human,mouse*	*Gapdh*	F: 5’-TGAACGGGAAGCTCACTGG-3’ R: 5’-TCCACCACCCTGTTGCTGTA-3’	

### 2.14 Immunofluorescence

The proximal small intestine and colon were surgically removed from the euthanized mice and flushed with phosphate-buffered saline. Tissues were fixed in 10 N Mildform (Wako), dehydrated using 20% sucrose, frozen in SCEM (Section Lab, Kanagawa, Japan), and sectioned at 6-µm thickness. Sections were incubated for 1 h with 0.2% Triton X-100 and 3% bovine serum albumin after antigen retrieval with 10 mM sodium citrate for 10 min at 95°C. They were then incubated overnight at 4°C with the primary antibodies: anti-GABA B receptor 1 (1:1000, #ab55051, Abcam, Cambridge, UK) or anti-GABA B receptor 2 (1:500, #27567-1-AP, Proteintech, Rosemont, IL, USA) and for 1 h with Alexa 647 (for GABA_B1_R) or Alexa 488 (for GABA_B2_R)-labeled secondary antibodies (1:400, Biolegend, CA, USA). DAPI (Invitrogen) was used for nuclear staining. Sections were stored in the dark at 4°C until they were imaged using confocal laser scanning microscopy (TCS SP8 LIGHTNING, Leica, Wetzlar, Germany).

### 2.15 Western blotting

Caco-2, HCT116, and murine IECs and organoids were suspended in lysis buffer (TBS pH 7.0, 1% Nonidet P-40, 60 mM n-octyl-ß-D-glucoside, 1% protease inhibitor cocktail (Nacalai Tesque, Kyoto, Japan), 1% phosphatase inhibitor cocktail (Nacalai Tesque), and 1 mM EDTA) and incubated on ice for 25 min. After centrifugation at 14,000 rpm at 4°C for 10 min, the supernatants were collected, and protein concentrations were measured using the Pierce™ BCA protein assay kit (Thermo Fisher Scientific). Equal amounts of protein were analyzed by immunoblotting with primary antibodies against p44/42 mitogen-activated protein kinase (MAPK) (extracellular signal-regulated kinase 1/2 (Erk1/2)), p-p44/42 MAPK (Erk1/2), stress-activated protein kinase/c-Jun N-terminal kinase (JNK), p-JNK, p38 MAPK, and p-p38 MAPK (#4695, #4370, #9252, #4668, #9212, and #4511, Cell Signaling Technology, Danvers, MS, USA). Lysates prepared from the intestinal organoids at the same passage were subjected to immunoblotting.

### 2.16 Statistical analysis

Statistical analyses were performed using a two-tailed Student’s t-test or one-way ANOVA, followed by Dunnett’s or Tukey’s tests. Differences with P< 0.05 were considered significant.

## 3 Results

### 3.1 Intestinal GABA concentration depends on the composition of gut microbiota

To test whether the alteration in microbiota composition affects intestinal GABA concentration, we first treated the mice with ampicillin, metronidazole, vancomycin, or neomycin (**Figure 1A**). Principal coordinate analysis showed that treatments resulted in different compositional changes in the gut microbiota for each antibiotic (**Figure 1B**). Intestinal GABA was quantified using feces to observe changes in concentration during the experiment. The fecal GABA concentration increased significantly after 14-day treatment with neomycin and remained higher than that of the control group on days 21 and 28. No remarkable changes were observed in the other treatment groups, although the metronidazole-treated group showed a slight increase in fecal GABA concentration after 28 days of treatment (**Figure 1C**). The neomycin and metronidazole groups had relatively similar gut microbiota compared to the other groups after the completion of treatment, but the composition of their microbiota was distinctly different (**Supplementary Figure 1**). Neomycin treatment did not alter the total amount of 16S rDNA in the feces (**Figure 1D**), confirming that it caused a change in the gut microbial composition without affecting the total amount of bacteria. Furthermore, serum GABA concentration did not increase during the neomycin treatment (**Figure 1E**), indicating that the increase in intestinal GABA concentration in the neomycin-treated group was due to its production by intestinal bacteria. These data suggest that intestinal GABA concentrations depend on the gut microbiota composition and can be modulated by interventions in the microbiota.

### 3.2 Administration of TMC3115 increases intestinal GABA concentration and alleviates host anxiety behavior

We next investigated whether intervention in the gut microbiota by administration of the probiotic bacterium TMC3115, which was isolated from healthy infant stool, affects intestinal GABA concentration and host behavior (**Figure 2A**). Because probiotic bacteria generally do not sustainably colonize the intestine for long periods, TMC3115 was repeatedly administered every 2 days during the experiment. As shown in **Figure 2B**, TMC3115 was not detected before administration (day 0) but was continuously detected from day 2 to day 14 in the feces of the TMC3115 group. TMC3115 was not detected in the feces of the control group during the experimental period. In addition, TMC3115 was detected in the cecal contents of the TMC3115 group but not in the control group two weeks after administration. These results confirmed that TMC3115 reached the intestine and was maintained at elevated levels shortly after administration. Fecal GABA concentrations did not change until day 6 but increased significantly on day 13 in the TMC3115 group, indicating that continued administration of TMC3115 elevated intestinal GABA concentrations (**Figure 2C**). In contrast, serum GABA concentration was not altered by the administration of TMC3115 for two weeks (**Figure 2D**). The fecal and serum GABA concentrations were not altered in the control group. We selected one strain each from the typical probiotics Bifidobacterium and Lactobacillus and used LGG in addition to TMC3115 for this study. However, LGG did not affect the fecal GABA concentrations, suggesting that this effect was dependent on the probiotic strain (**Supplementary Figure 2**).

To examine whether the increase in intestinal GABA following TMC3115 administration affected host behavioral characteristics, an elevated plus maze test was conducted. TMC3115 treatment reduced anxiety-like behavior, as shown by the increased entries into the open arms and unaffected entries into the closed arms (**Figure 2E**). Although corticosterone and serotonin levels are known to be involved in anxiety-like behavior, the administration of TMC3115 did not affect serum corticosterone, serotonin, or fecal serotonin levels (**Figure 2F**). The effect of TMC3115 treatment on spatial cognitive memory was also assessed using y-maze test. TMC3115 treatment slightly increased the alternation rate and slightly decreases the locomotor activity but there is no significant difference (**Supplementary Figure 3**).

Since TMC3115 does not possess GABA synthesis enzyme genes (Gotoh et al., 2018), the elevated intestinal GABA level in the TMC3115 group is presumably due to the change in gut microbiota composition, i.e., an increase in bacteria with high GABA-producing capabilities or a decrease in those with high GABA-consuming capabilities. Analysis of the changes in microbiota composition after administration of TMC3115 showed that *Lactobacillus* and *Clostridium* bacteria increased and decreased, respectively, at both the order and genus levels (**Figures 2G, H**). Collectively, these data suggest that the administration of TMC3115 increases the intestinal GABA concentration, probably through changes in the microbiota composition, resulting in behavioral alterations.

### 3.3 IECs express GABARs

**Figure 3:**
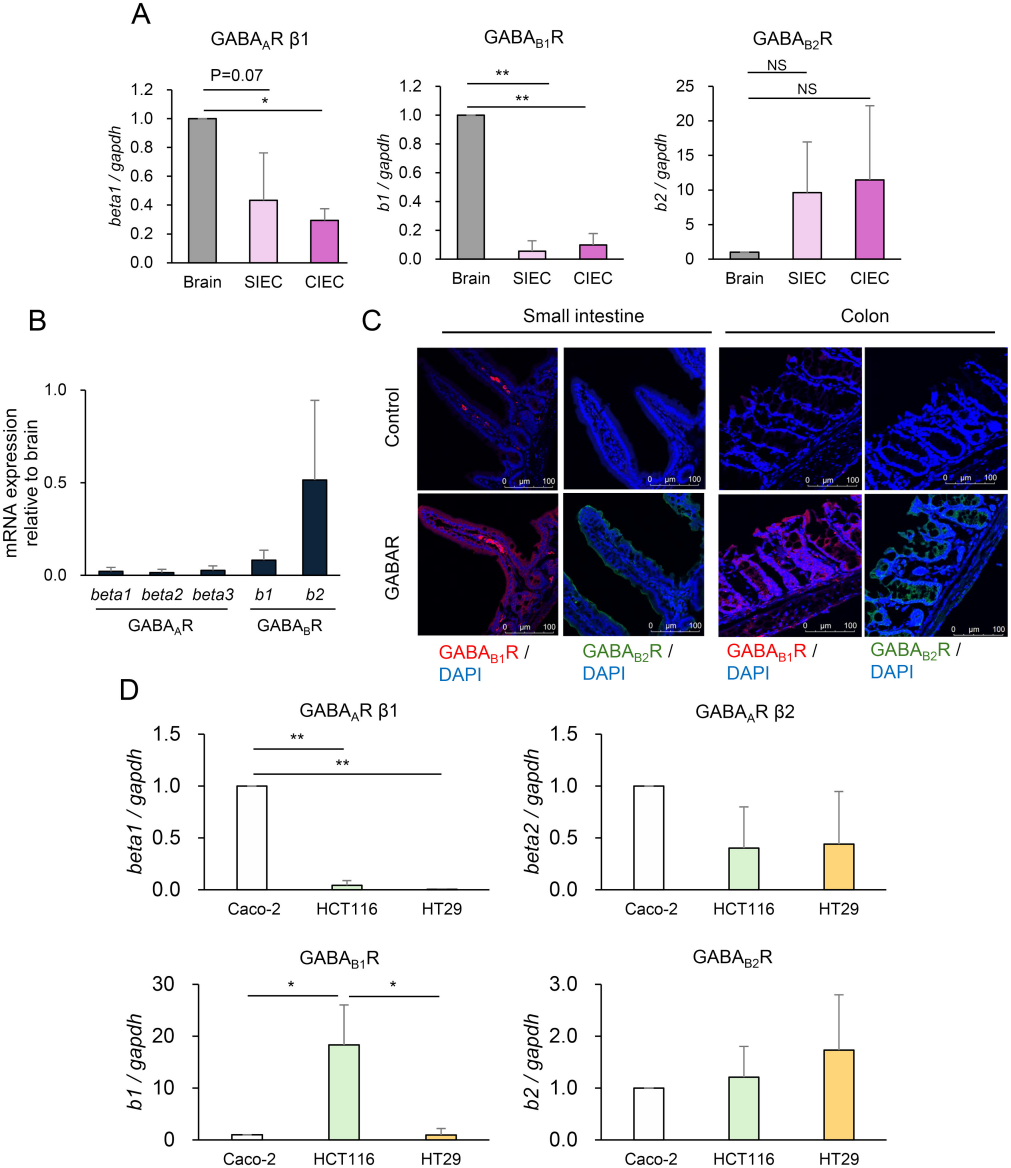
Intestinal epithelial cells (IECs) express γ-aminobutyric acid receptor (GABAR). **(A)** Expression of GABAR subunits GABA_A_R β1 (left), GABA_B1_R (middle), and GABA_B2_R (right) in IECs prepared from the small intestine (SIECs), IECs prepared from the colon (CIECs), and the brain of mice was quantified using RT-qPCR. Expression levels relative to that in the brain are presented as mean ± SD of three independent experiments. NS, not significant; *P <0.001; **P <0.0001. **(B)** Expression of GABAR subunits GABA_A_R β1, GABA_A_R β2, GABA_A_R β3, GABA_B1_R, and GABA_B2_R in mouse intestinal organoids and brain was quantified using RT-qPCR. Expression levels relative to those in the brain are presented as mean ± SD of eight independent experiments. **(C)** GABAR expression was analyzed in mouse small intestinal and colonic tissue sections using immunofluorescence. Representative confocal images of GABA_B1_R (left) and GABA_B2_R (right) in the small intestine and colon are shown. Top, isotype control or without primary antibody; bottom, anti-GABA_B_R antibody. **(D)** Expression of GABAR subunits GABA_A_R β1, GABA_A_Rβ2, GABA_B1_R, and GABA_B2_R in human IEC lines Caco-2, HCT116, and HT-29 was quantified using RT-qPCR. Expression levels relative to those in Caco-2 are presented as mean ± SD of three independent experiments. *P < 0.05; **P < 0.001.

Based on the finding that elevated intestinal GABA concentrations affect host behavior without altering serum GABA concentrations, we hypothesized that GABA produced by commensal bacteria primarily in the colon, causes behavioral changes through its action on IECs that are directly exposed to the luminal contents. GABAR mRNA expression was analyzed quantitatively using IECs prepared from the mouse small intestine and colon (SIECs and CIECs, respectively) and compared with its expression in the brain, where it has been proven to be functional. GABA_A_R is a pentamer composed of various combination of α1-6, β1-3, γ1-3, δ, ϵ, π, θ, and ρ subunits, of which β1-3 was measured as the β subunit is essential for GABA binding along with α subunit. GABA_B_R is expressed as a heterodimer consisting of GABA_B1_R and GABA_B2_R. SIECs and CIECs expressed GABA_A_R β1, GABA_B1_R, and GABA_B2_R (**Figure 3A**). Surprisingly, GABA_B2_R expression was much higher in the SIECs and CIECs than in the brain. GABA_A_R β2 and GABA_A_R β3 were almost undetectable in SIECs and CIECs (data not shown). The expression of multiple GABAR subunits was also detected in mouse intestinal organoids, of which GABA_B2_R expression was comparable to that in the brain, whereas the expression of other subunits was lower than that in the brain (**Figure 3B**). Furthermore, immunofluorescence analysis of the mouse small intestinal and colonic tissues showed the expression of GABA_B1_R and GABA_B2_R in the epithelial layer, in addition to the periglandular area in the colon and some cells in the small intestinal lamina propria (**Figure 3C**). The expression levels of epithelial GABA_B1_R and GABA_B2_R were similar in the small intestine and colon, which was consistent with their comparable mRNA expression levels in SIECs and CIECs (**Figure 3A**). These results suggest that the intestinal epithelia express GABARs, especially GABA_B_R, as the predominant type, suggesting that IECs are highly responsive to luminal GABA via GABA_B_R.

### 3.4 GABA induces MAPK signaling in IECs

**Figure 4:**
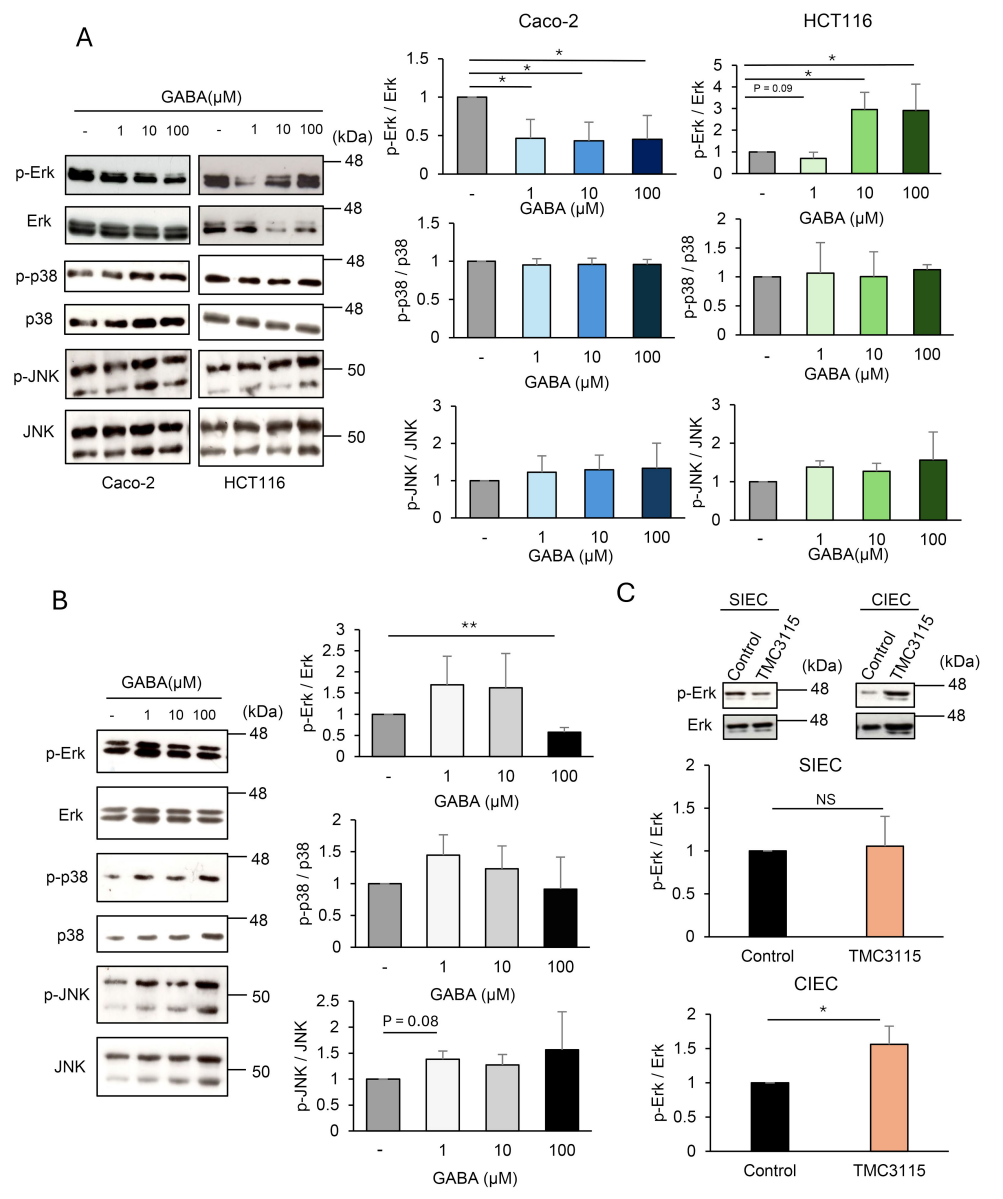
Intestinal epithelial cells (IECs) are responsive to γ-aminobutyric acid (GABA). **(A)** Mitogen-activated protein kinase (MAPK) phosphorylation in Caco-2 and HCT116 stimulated with GABA for 30 min was analyzed using western blotting. Representative blots (left) and mean ± SD of phosphorylation levels relative to control without GABA stimulation (right) from three independent experiments are shown. Phosphorylation levels were calculated by dividing the band intensities of phosphorylated molecules by those of the corresponding total molecules. *P < 0.05. **(B)** MAPK phosphorylation in intestinal organoids stimulated with GABA for 30 min was analyzed using western blotting. Representative blots (left) and mean ± SD of phosphorylation levels relative to control without GABA stimulation (right) from three independent experiments are shown. Phosphorylation levels were calculated by dividing the band intensities of phosphorylated molecules by those of the corresponding total molecules. **P < 0.01. **(C)** Erk phosphorylation in SIECs and CIECs isolated from *Bifidobacterium bifidum* TMC3115-administered mice (n=10) and control mice (n=10) were analyzed using western blotting. Representative blots (top) and mean ± SD of phosphorylation levels relative to control mice (bottom) are shown. Lysates from pooled IECs for 4-6 mice each were analyzed. Phosphorylation levels were calculated by dividing the band intensities of phosphorylated molecules by those of the corresponding total molecules. NS, not significant; *P < 0.05.

Next, we examined whether GABA triggers intracellular signaling in IECs using the human colonic epithelial cell lines Caco-2, HCT116, and HT-29. These IEC lines were confirmed to express GABAR, with Caco-2 and HCT116 cells predominantly expressing GABA_A_R and GABA_B_R, respectively (**Figure 3D**). MAPK signaling upon GABA stimulation was evaluated in Caco-2 and HCT116 cells (**Figure 4A**). Erk was significantly inhibited by GABA in Caco-2 cells but was significantly activated in HCT116 cells. p38 showed no changes, and JNK was slightly activated by GABA stimulation in both cell lines. These results indicated that GABA elicited MAPK signaling in IECs and that GABAergic signals differed depending on the type of GABARs expressed. GABAergic signaling was further investigated in mouse intestinal organoids. MAPK signaling was also induced by GABA stimulation in intestinal organoids. Erk was activated by GABA stimulation, and p38 and JNK also tended to be activated (**Figure 4B**). The signaling pattern was similar to that of HCT116 cells, which also predominantly expressed GABA_B_R, although a different dose-dependence was observed for Erk activation. The effect of TMC3115 administration in mice on Erk phosphorylation in IECs was further analyzed (**Figure 4C**). TMC3115 administration upregulated Erk phosphorylation in CIECs but not in SIECs, suggesting that GABA produced by the gut microbiota may directly act on IECs in the colon, where most gut microbes exist.

Collectively, these results indicate that GABA may act directly on IECs to induce intracellular signaling, thereby affecting the functional and/or structural properties of the intestinal epithelia, which may lead to behavioral changes.

## 4 Discussion

In this study, we identified the potential of intestinal GABA to influence host behavioral characteristics via GABARs expressed in IECs, revealing not only the physiological significance of intestinal GABA but also a novel mechanism for its action. Intervention in the gut microbiota increased intestinal GABA levels and reduced anxiety behavior, indicating that intestinal GABA may be a target for the prevention and treatment of psychiatric and neurological disorders with probiotics. The roles of the gut microbiota in these disorders have been highlighted from the perspective of the gut-brain axis in recent years, and our study suggests a new role through the production of GABA. Orally administered GABA shows various physiological effects despite the presence of the BBB (Abdou et al., 2006; Nakamura et al., 2009; Yoto et al., 2012), suggesting the possibility that GABA acts through an unknown pathway independent of blood circulation or GABARs in the brain. TMC3115 administration increased intestinal GABA but did not affect serum GABA levels (**Figures 2C, D**), indicating that the reduction in anxiety behavior was not mediated by GABA transferred from the intestinal tract into the blood. We found that IECs express GABAR and that GABA stimulation induced MAPK signals in IECs (**Figures 3**, **4**), suggesting a possible mechanism that works through IECs regardless of the presence of the BBB. Because most gut microbes inhabit the colon, GABA is thought to be produced by the gut microbiota primarily in the colon, which is consistent with the finding that Erk was activated in CIECs but not in SIECs when TMC3115 was administered (**Figure 4C**). This may account for the fact that GABA derived from the gut microbiota is hardly absorbed in the small intestine and is rarely transferred into the blood, unlike GABA derived from foods. Gut microbiota- and food-derived GABA may function via different mechanisms. Future studies should determine the GABA concentrations in the small intestinal and colonic lumen to clarify the intestinal portion-specific action of GABA. Another important aspect is the elucidation of the mechanisms underlying the action of GABA via GABARs on IECs. MAPK signals elicited in IECs may affect IEC function and influence the epithelial barrier and permeability, the disruption of which is known to result in various diseases, including neurological and psychiatric disorders. These resulting effects may be mediated by gut-derived neurotransmitters or metabolites other than GABA, which pass through the BBB and enter the brain. Another possibility is that intestinal GABA transmits GABAergic signals to the brain via the vagus nerve, as dietary GABA has been reported to regulate feeding behavior by activating the vagal afferent nerves (Nakamura et al., 2022). Alternatively, both IEC-mediated and vagus nerve-mediated mechanisms may be involved. Further studies, including investigations of brain regions associated with anxiety, are needed to understand the mechanisms underlying the action of intestinal GABA. In addition, behavioral changes induced by intestinal GABA need to be evaluated more precisely by combining other tests with the elevated plus maze test.

The human IEC lines Caco-2 and HCT116 showed different expression patterns of GABAR, and GABA induced different MAPK signals, depending on the type of GABAR expressed (**Figures 3D**, **4A**). The inhibition of GABA_A_R ameliorated intestinal mucosal tissue damage in a dextran sulfate sodium-induced colitis mouse model (Ma et al., 2018), and the activation of GABA_B_R inhibited colorectal cancer cell proliferation (Song et al., 2016), which implies that GABA might have different effects through different GABAR pathways. Mouse SIECs, CIECs, and intestinal organoids expressed GABA_B2_R as abundantly as the brain, leading to the hypothesis that the GABA_B_R pathway primarily functions in steady-state IECs. The induction of Erk activation observed in CIECs of TMC3115-treated mice (**Figure 4C**) was likely mediated through the GABA_B_R pathway, as similar Erk activation was detected in HCT116 cells and intestinal organoids that predominantly express GABA_B_R, but not in Caco-2 cells, which predominantly expresses GABA_A_R (**Figures 4A, B**). Increasing evidence has shown that exosomes derived from GABA-treated Caco-2 cells activate neuronal cells by regulating genes related to neuronal cell function, although GABAR dependence has not been reported (Inotsuka et al., 2020). Detailed signal transduction via GABARs in the IECs requires further investigation, but the GABAergic signal may alter functional and structural properties of IECs, thereby affecting behavioral characteristics, as various studies have reported an association between leaky gut and behavioral changes in IBD patients (Ge et al., 2022). LGR5^+^ intestinal epithelial stem cells express GABA_A_R, and the depletion of GABA_A_R β1 has been reported to ameliorate cell apoptosis during chemoradiotherapy (Zhang et al., 2022). Another study showed that GABA_B_R-mediated signaling induces apoptosis through the inhibition of cAMP signaling pathways in colorectal cells undergoing chemotherapy (An et al., 2021), suggesting that GABAergic signaling plays a role in oncogenic IEC proliferation. Although the role of GABAergic signaling in normal IECs remains unclear, intestinal GABA may regulate IEC properties through GABAR to affect its function. GABA may also affect mucus secretion in the colonic epithelium, as GABA_B_R was expressed in the periglandular area in addition to the epithelial layer (**Figure 3C**).

TMC3115 administration increased and reduced the abundance of Lactobacillales and Clostridiales in the mouse cecum, respectively (**Figure 2G**). Since TMC3115 does not possess GABA synthesis enzyme genes (Gotoh et al., 2018), it is speculated that the increase in intestinal GABA levels results from the proliferation of bacteria with high GABA-producing capabilities or the reduction in those with high GABA-consuming capabilities. Some Lactobacillus strains, such as those belonging to *Levilactobacillus brevis* and *Lactiplantibacillus plantarum*, possess *gad* genes and produce GABA from glutamate (Matta et al., 2024; Diez-Gutiérrez et al., 2022). Conversely, although there are few reports demonstrating Clostridium’s ability to consume GABA, *Clostridium aminobutyricum* has been reported to ferment GABA to ammonia, acetate, and butyrate (Macieira et al., 2012). Interestingly, in a recent study showing the correlation between the likelihood of developing autism spectrum disorder and early life intestinal GABA concentration, infants with elevated-likelihoods had fewer *Bifidobacterium* and *Lactobacillus*, and more *Clostridium* species in their gut and lower levels of fecal GABA at five months of age than those with low-likelihoods, suggesting the physiological role of intestinal GABA and the involvement of these bacteria (Zuffa et al., 2023). TMC3115 was detected from day 2 of administration, whereas an increase in intestinal GABA was observed on day 14, suggesting that TMC3115 alters the intestinal microbiota composition with some delay after colonization. TMC3115 possesses numerous genes that encode enzymes involved in the degradation of human milk oligosaccharides (HMOs), which are nutrients for intestinal bacteria. *In vitro* co-cultivation of human fecal samples with HMOs and TMC3115 has been reported to increase the abundance of other *Bifidobacterium* species in the culture medium during HMO degradation (Gotoh et al., 2018). Thus, TMC3115 may affect the composition of the gut microbiota by providing a nutrient source available to other intestinal bacteria in the intestinal tract.

Previous studies have reported that TMC3115 and LGG promote the function and development of neurons via increased expression of brain-derived neurotrophic factor *in vitro* (Cheng et al., 2020). However, in our study, the effects on behavioral characteristics were observed only in mice administered TMC3115 and not in those administered LGG. This may reflect that the effects of TMC3115 are mediated by changes in the gut microbiota, that is, the proliferation of GABA-producing bacteria, rather than the direct effects of TMC3115 itself. It is also possible that the bacterial components of TMC3115 directly affect neurons and cooperate with intestinal GABA to modulate host behavior. The dependence of the behavioral changes observed in TMC3115-treated mice on GABARs in IECs must be further evaluated. We do not deny the involvement of mechanisms other than intestinal GABA in the behavioral effects of TMC3115. Because the administration of TMC3115 increased the concentration of cecal butyrate (**Supplementary Figure 4**), butyrate may affect the IEC function in concert with intestinal GABA. Butyrate is well known to exert a variety of physiological effects, including those on intestinal epithelium maintenance and BBB integrity (Joyce and Clarke, 2024; Chakraborty et al., 2024), and thus may affect the gut-brain homeostasis in a coordinated manner with GABA. The decrease in the abundance of bacteria belonging to the order Clostridiales, known as butyrate-producing bacteria, after TMC3115 administration suggests that TMC3115 administration selectively increases the number of specific bacteria within the order Clostridiales that produce high levels of butyrate or reduces butyrate-consuming bacteria.

Our findings demonstrate that intestinal GABA levels depend on gut microbiota composition and can affect host behavior, providing a novel mechanism of action via the intestinal GABA-IEC axis.

## Data Availability

The datasets presented in this study can be found in online repositories. The names of the repository/repositories and accession number(s) can be found in the article/**Supplementary Material**.
