# Supplementary material for: Intervention in gut microbiota increases intestinal γ-aminobutyric acid and alleviates anxiety behavior: a possible mechanism via the action on intestinal epithelial cells
Source: Front Cell Infect Microbiol. 2024 Sep 5;14:1421791. doi: 10.3389/fcimb.2024.1421791 (PMC11410766; doi:10.3389/fcimb.2024.1421791)
Supplement: Supplementary file 1 [file DataSheet1.pdf]

Supplementary Material

1 Supplementary Data

1.1 Y-maze test

Spatial working memory and spontaneous locomotor activities were measured using a Y-maze apparatus consisting of a three-arm horizontal maze (41.5 cm length, 4 cm width, 10 cm height; arms symmetrically positioned at 120° angles from the center area). The mice were placed in one arm and allowed to explore freely. The sessions lasted for 6 min for recording. The number of entries in each arm was counted by tracking the position of the mouse using the ANYMAZE software (Stoelting). Spontaneous locomotor activity was defined as the total number of arm entries. The percentage of alteration behavior (entries to arms other than the one entered just before), calculated using the following formula, was used as an index for spatial working memory: {(Number of alterations) / (Total arm entries - 2)} ×100.

1.2 Determination of butyric acid concentration in cecum contents

Determination of butyric acid concentration in cecum contents using the GC-MS system (GC-2010, GC-MS-QP2010, Shimadzu, Kyoto, Japan) equipped with DB-23 column (0.25 mm × 60 m, 0.15 µm, Agilent, Santa Clara, CA, USA). Samples were methyl esterified, including a series of serial dilutions of methyl butyrate solution in the range of 0.125 to 200 µg/mL for the calibration curve. The concentration of butyric acid per 1 mg of cecum content was calculated.

2 Supplementary Figures

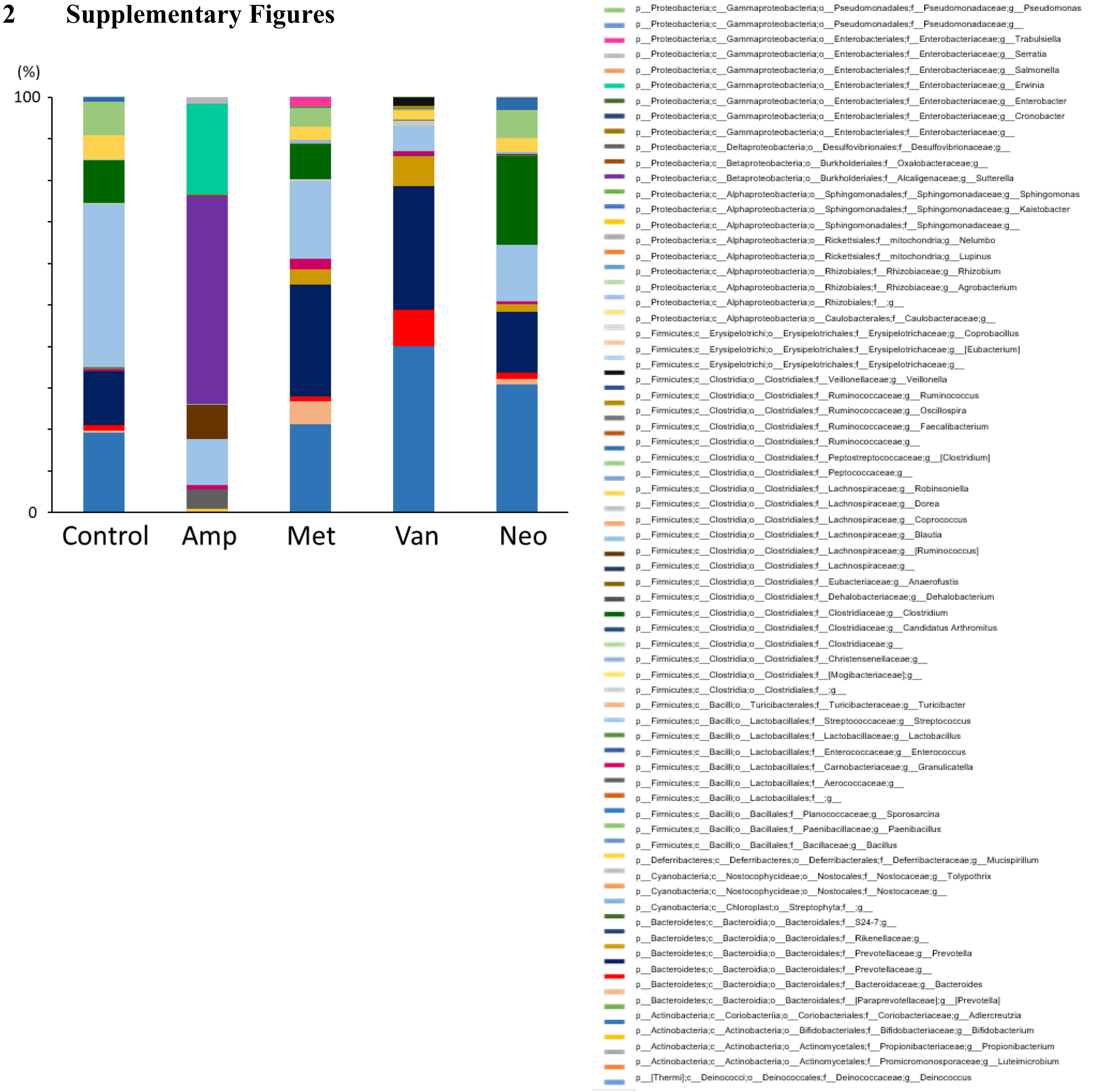

Supplementary Figure 1. Different antibiotics cause different gut microbiota changes. Gut microbiota in Amp, Met, Van, and Neo-treated and untreated (Control) mice were analyzed by 16S rRNA sequencing using fecal DNA pooled from 3 mice/group on day 28. The microbiota composition at the genus level is shown as percentages in the stacked bar chart.

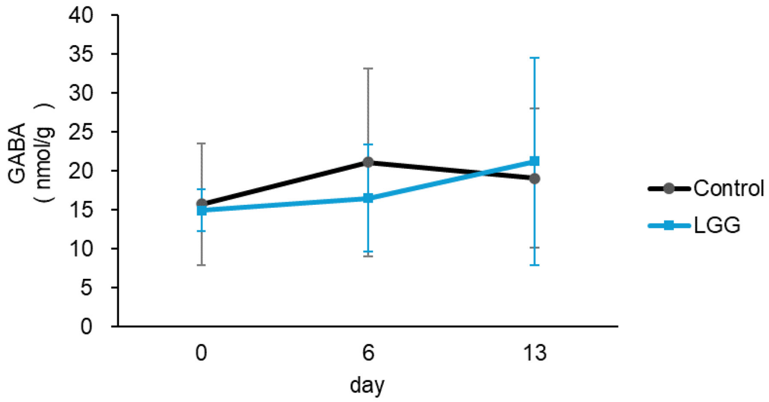

Supplementary Figure 2. LGG does not increase the intestinal GABA. Lyophilized LGG was suspended in saline and administered orally at 10^9 CFU/head/day every other day for two weeks. Fecal GABA concentration was measured using UPLC-MS/MS system on days 0, 6, and 13. Data are presented as mean ± SD (Control, n=16; LGG, n=9).

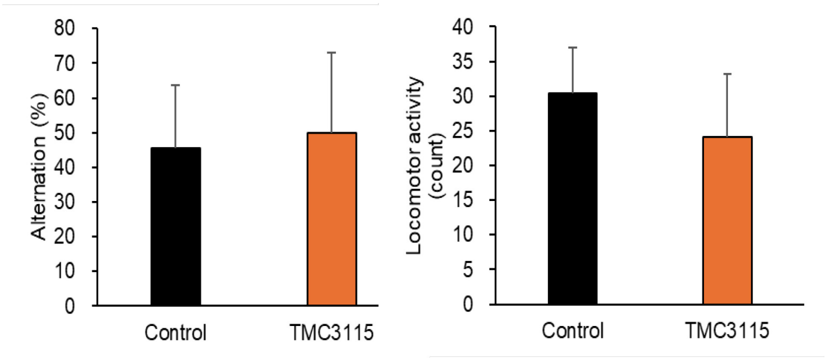

Supplementary Figure 3. Behavioral analysis using Y-maze. Y-maze test was performed in control and TMC3115-treated mice. Data are presented as mean ± SD (Control, n=10; TMC3115, n=10).

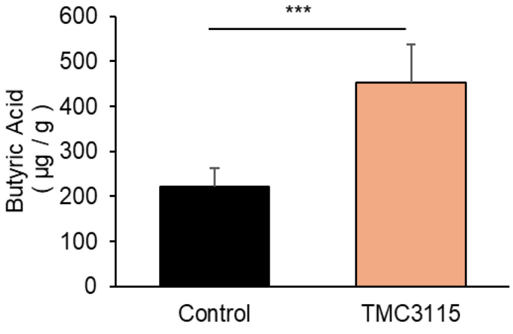

Supplementary Figure 4. TMC3115 administration increases butyric acid in the intestine. Cecal butyric acid concentration was determined in TMC3115-treated and control mice using GC-MS. Data are presented as mean ± SD (Control, n=5; TMC3115, n=5). ***, P < 0.0005.
